# Cord blood therapy for pure red cell aplasia after allogeneic hematopoietic stem cell transplantation: case series and review

**DOI:** 10.3389/fonc.2025.1585088

**Published:** 2025-06-03

**Authors:** Zhen Li, Sujing Zhuang, Ruirui Gui, Binglei Zhang, Wenli Zhang, Juan Wang, Yingling Zu, Fei Yang, Xiangke Xin, Yanyan Liu, Yanli Zhang, Baijun Fang, Fengkuan Yu, Huifang Zhao, Wei Li, Yongping Song, Jian Zhou

**Affiliations:** ^1^ Department of Haematology, Affiliated Cancer Hospital of Zhengzhou University and Henan Cancer Hospital, Zhengzhou, Henan, China; ^2^ Shandong Qilu Stem Cell Engineering Co. LTD., High-tech Development Zone, Jinan, Shandong, China; ^3^ Department of Haematology, The First Affiliated Hospital of Zhengzhou University, Zhengzhou, Henan, China; ^4^ Department of Oncology, Anyang People’s Hospital, Anyang, China

**Keywords:** pure red cell aplasia, allogeneic hematopoietic stem cell transplantation, cord blood, mesenchymal stem cells, cell therapy

## Abstract

**Introduction:**

Pure red cell aplasia (PRCA) is one of the complications after allogeneic hematopoietic stem cell transplantation (allo-HSCT). Its main pathogenesis is immune dysfunction leading to erythrocytes destruction. Currently, there is no gold standard for PRCA after allo-HSCT. Umbilical cord blood (UCB) and mesenchymal stem cells (MSCs) have been widely used in hematological and immune system diseases due to their hematopoietic reconstitution and immunomodulatory functions. However, few studies about using UCB and MSCs to treat PRCA after allo-HSCT have been reported.

**Case presentation:**

In this report, different cell therapy regimens of UCB and MSCs were used in 3 acute myeloid leukemia (AML) patients diagnosed with PRCA after allo-HSCT. Results showed that all patients achieved significant progress without adverse reactions or complications. Furthermore, Case 1 treated with UCB combined with umbilical cord MSCs (UC-MSCs), and Case 2 treated with 3 doses of UCB mononuclear cells (UCB-MNC) achieved earlier RBC transfusion independence (2 months and 2 weeks after cell therapy, respectively) than Case 3 treated with one unit of UCB (3 months after cell therapy).

**Conclusion:**

This report provides cell therapy strategies using UCB/UCB-MNC and UC-MSCs to treat PRCA after allo-HSCT. Our study demonstrates the safety and efficacy of 3 doses of UCB-MNC regimen and UCB combined with UC-MSCs regimen, providing a new treatment option for patients with PRCA after allo-HSCT.

## Introduction

Pure red cell aplasia (PRCA) is one of the complications in allogeneic hematopoietic stem cell transplantation (allo-HSCT), especially in patients undergoing ABO blood group-incompatible HSCT, and the incidence of PRCA after allo-HSCT is approximately 7%-30% (1–4). PRCA could cause delayed recovery of erythropoiesis and many complications, such as blood transfusion dependence, iron overload, and secondary infection (5, 6).

PRCA after allo-HSCT was characterized by anemia, low reticulocyte (RET) counts (<1%) in peripheral blood for more than 60 days after allo-HSCT, and a lack of erythroid precursors in bone marrow (7). The pathogenesis is still unclear, mainly caused by the interaction between donor-derived red blood cells and residual or persistent allogenic antibodies in the recipient, anti-donor isohemagglutinins (IH) were produced in the patient, which could mediate immune abnormalities and lead to the destruction of red blood cells and erythroid precursor cells in donors (3).

There is currently no “gold standard” to treat PRCA after HSCT (8). Many therapies have been used to treat PRCA, including erythropoietin (EPO), desensitization apheresis (plasma exchange, rituximab, and etc.), immunosuppression, and donor leukocyte infusion (DLI), but the efficacy is different and unsatisfactory (2, 9, 10). With the development of cell therapy, UCB and MSCs have been increasingly used in hematological and immune system diseases due to their hematopoietic supporting and immunomodulatory functions. Moreover, UCB has the advantage of a lower probability of causing PRCA after allo-HSCT (1, 10–12). However, studies about using UCB and MSCs to treat PRCA are exceedingly infrequent, especially in PRCA after allo-HSCT (10, 13–15).

In this study, we reported three cell therapy regimens in PRCA after allo-HSCT, including UCB combined with MSCs, repeated doses of UCB-MNC, and one unit of UCB. The protocol and results would provide data support for the choice of treatment regimens for PRCA after allo-HSCT.

## Case presentation

### Case 1

**Figure 1:**
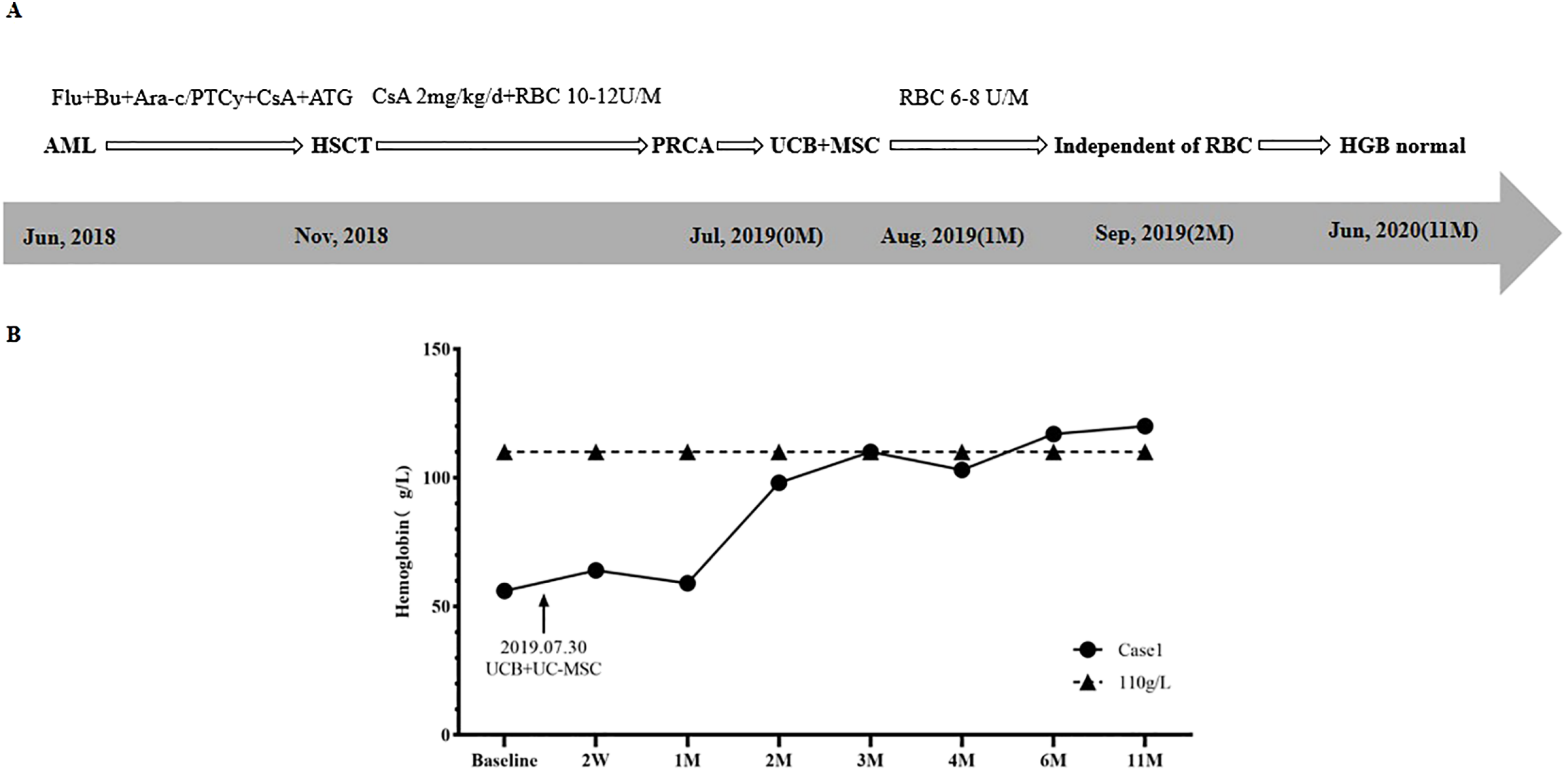
Treatment regimen **(A)** and changes of HGB **(B)** in case 1.

A 32-year-old female was diagnosed with acute myeloid leukemia (AML) in June 2018. As shown in **Figure 1**, in November 2018, the patient underwent HLA-identical sibling peripheral blood hematopoietic stem cell transplantation (HSCT). The blood types of the patient and donor were O/Rh+ and B/Rh+, respectively. Anti-A IgM and anti-B IgM titers were 1:64 and 1:128, respectively. A conditioning regimen of Fludarabine (FLU) + Busulfan (BU) + Cytosine Arabinoside (Ara-C) was used. A total of 11.56×10^8^/Kg mononuclear cells (MNC) (including 8.9×10^6^/Kg CD34+ cells) were infused. The Graft-versus-host disease (GvHD) prevention regimen was Posttransplant cyclophosphamide (PTCy) combined with anti-thymocyte globulin (ATG) and Cyclosporine A (CsA). On the 11^th^ and 12^th^ days after transplantation, the patient achieved megakaryocyte and granulocyte lineages recovery.

One month after HSCT, the patient’s physical examination (with an anemic face) and blood routine test (BRT) showed anemia, with HGB of 65 g/L and RET of 1.2×10^9^/L (0.1%), so she received intermittent red blood cell (RBC) transfusion therapy. Results of bone marrow aspirate smear (BMA) showed that granulocyte cells and erythroid cells accounted for 88.6% and 0.0%, respectively. Short tandem repeat (STR) chimerism analysis showed that donor cells accounted for 99.23%. No infection of Epstein-Barr virus (EBV), Cytomegalovirus (CMV), Human parvovirus B19 (HPV B19), or hemorrhagic cystitis (HC) and GvHD occurred. The patient was considered to be PRCA. Supportive therapy was taken for 6 months, including 2mg/kg/d CsA and 10U-14U/month of RBC. However, the RET remained lower than 1% (0.1%-0.2%), and the patient was dependent on RBC transfusion (HGB < 60g/L).

In July 2019, BRT showed that WBC was 2.95×10^9^/L, HGB was 56g/L, and RET was 0.15%. BMA results displayed that granulocyte cells accounted for 80.0%, erythroid cells accounted for 0.0%, and megakaryocytes could be seen. The patient was diagnosed with PRCA after allo-HSCT.

The treatment regimen and change of HGB after the diagnosis of PRCA are displayed in **Figure 1**. On July 30^th^, 2019, one unit of UCB (with 6/10 HLA compatibility by high resolution) and 100mL UC-MSCs were infused. The UCB unit was from Shandong Qilu Stem Cell Engineering Co., LTD, of which total nucleated cells (TNC) was 20.83×10^8^ (including 4.20×10^6^ CD34+ cells), and UC-MSCs was 6.0×10^7^. As shown in **Figure 1B**, 2 weeks (2W) after cell infusion, HGB increased to 64g/L; one month (1M) after cell infusion, HGB remained stable (59 g/L), but the RBC transfused decreased from 10U-14U/month to 6U-8U/month. Two months (2M) after cell infusion, HGB rose to 98g/L, and the patient became independent of RBC transfusion.

Four months after cell infusion, the results of BMA showed that the proliferation of nucleated cells was significantly active, with 59.6% of granulocyte cells and 6% of erythroid cells, implying the erythroid cells were gradually recovering. Six months after cell infusion, BMA results showed granulocyte cells accounted for 36.4%, and erythroid cells accounted for 43.8%, indicating erythroid cells recovered to normal. Eleven months after cell infusion, HGB returned to 120g/L. Five years after HSCT (June 2024), HGB remained normal, and the patient’s primary disease was completely relieved.

### Case 2

**Figure 2:**
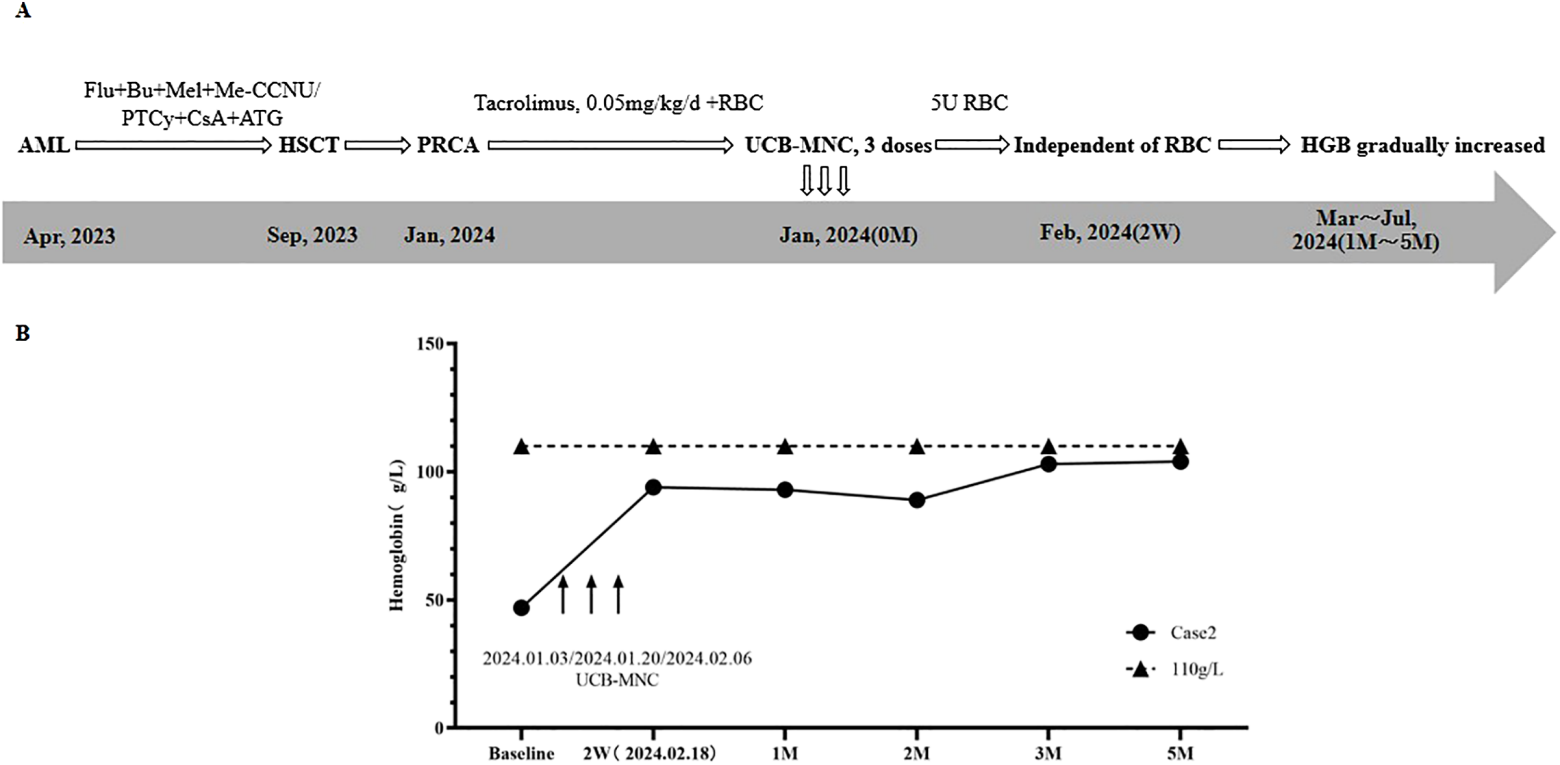
Treatment regimen **(A)** and changes of HGB **(B)** in case 2.

A 38-year-old female was diagnosed with AML in April 2023. Treatment regimens are shown in **Figure 2A**. In September 2023, HLA-identical sibling peripheral blood HSCT was performed. The patient’s blood type was O/Rh+, while the donor’s blood type was A/Rh+. Anti-A IgM and Anti-B IgM titers were both 1:128. The conditioning regimen was FLU+BU+melphalan (Mel) + Semustine (Me-CCNU), and a total of 8.28×10^8^/Kg MNC (5.88×10^6^/Kg CD34+ cells) were transfused. GvHD prevention regimen was PTCy combined with ATG and CsA.

Twelve days after HSCT, granulocyte and megakaryocyte lineages recovered. One month after HSCT, STR chimerism analysis showed that donor cells accounted for 98.56%. BRT showed that HGB was 52g/L, and RET was 0.8×10^9^/L (0.1%). Supportive therapy with intermittent RBC transfusion was used to improve anemia. Two months after HSCT, RET continued to be lower than 1%, and more RBCs were transfused. Three months after HSCT, BMA results displayed that granulocyte cells and erythroid cells accounted for 91.6%, and 3.2%, respectively. No infection of EBV, CMV, HPV B19, or HC and GvHD occurred. Based on the above results of examinations, the patient was diagnosed with PRCA after allo-HSCT.

In January 2024, the patient was admitted to the hospital due to severe anemia. The treatment regimen and change of HGB after the diagnosis of PRCA are shown in **Figure 2**. At first, Tacrolimus (0.05mg/kg/d) and supportive RBC transfusion were used, but there was no obvious improvement in HGB, and the amount of RBC infused per month gradually increased. From January 3^rd^ to February 6^th^, 2024, 3 doses of UCB-MNC were infused, with an interval of 17 days. During this period, 5U RBCs were also transfused. UCB-MNC was from Shandong Qilu Stem Cell Engineering Co., LTD, and the cell number was 5.0×10^7^ per dose.

As shown in **Figure 2B**, two weeks after the last UCB-MNC infusion, the patient’s HGB rose to 94 g/L, and the patient achieved RBC transfusion independence. One month after cell infusion, HGB was stable at 93 g/L. BMA results showed that granulocyte cells and erythrocyte cells accounted for 30.8% and 60.4%, respectively. The results of following-up from 2 months to 5 months after cell infusion showed that HGB gradually increased to 104 g/L and the primary disease was completely relieved. No GvHD, or EBV and CMV infection occurred.

### Case 3

**Figure 3:**
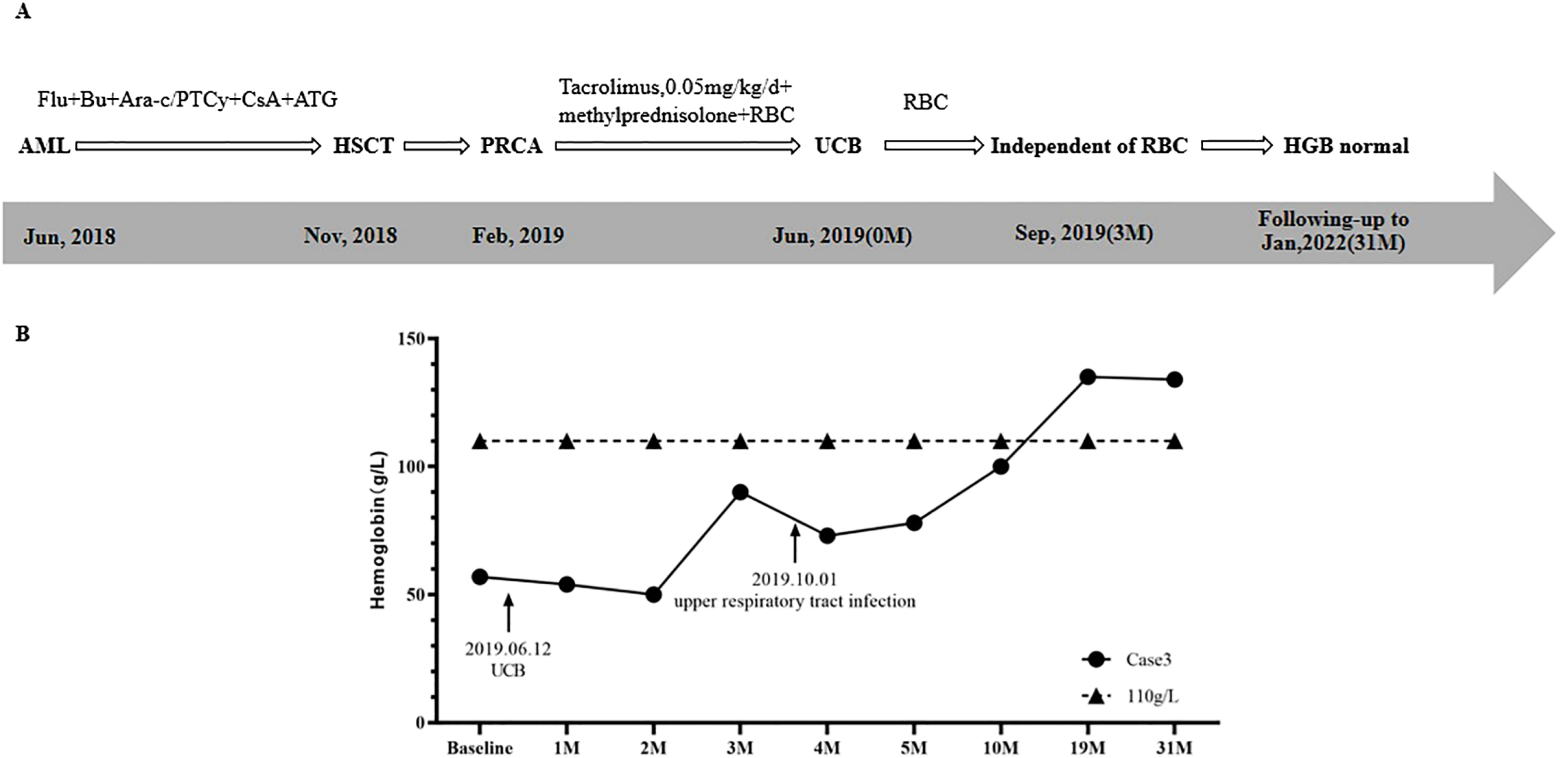
Treatment regimen **(A)** and changes of HGB **(B)** in case 3.

A 36-year-old female was diagnosed with AML in June 2018. Treatment regimens are shown in **Figure 3A**. In November 2018, HLA-identical sibling peripheral blood stem cell transplantation was performed. The patient’s blood type was O/Rh+, and the donor’s blood type was B/Rh+. The results of anti-donor IH titers of anti-A IgM and anti-B IgM were 1:32 and 1:16, respectively.

The conditioning regimen was FLU+BU+Ara-c, and a total of 26.60×10^8^/kg MNC with 8.3×10^6^/kg CD34+ cells were transfused. GvHD prevention regimen was PTCy combined with ATG and CsA. The granulocyte and megakaryocyte lineages were reconstructed on the 15^th^ day and 17^th^ day after transplantation, respectively. STR chimerism analysis showed donor cells accounted for 99.33%.

Three months after HSCT, in February 2019, the patient was admitted to hospital due to fatigue. Physical examination showed that the patient had an anemic face, and others were normal. BRT showed HGB was 49 g/L and RET was 0.3%. Results of BMA showed that granulocytes cells and erythroid cells accounted for 71.2% and 2%, respectively, while megakaryocytes were rarely found. A diagnosis of PRCA after allo-HSCT was made.

Interventions and changes of HGB are shown in **Figure 3**. After the diagnosis of PRCA, a supportive RBC transfusion was given. With CsA discontinued, methylprednisolone (8mg/time, 2 times/day) and tacrolimus (0.05mg/kg/day) were used to treat PRCA and prevent GvHD. However, after 4 months of treatment, the effect was not obvious. On June 12^th^, 2019, one unit of UCB (with 4/6 HLA compatibility by low resolution) was infused, of which TNC was 4.51×10^8^, and CD34+ cells was 2.56×10^6^. As shown in **Figure 3B**, HGBs from 1 month to 2 months after cell infusion were 54.0 g/L and 50.0 g/L, respectively. RBC transfusion was still needed to treat anemia. Three months after cell infusion, HGB recovered to 90.0 g/L, and the patient achieved RBC transfusion independence. On October 1^st^, the patient had upper respiratory tract infection, and HGB decreased slightly. After symptomatic treatment, HGB gradually increased. Ten months after cell infusion, HGB increased to 100.0 g/L. Nineteen months after cell infusion, HGB recovered to 135.0 g/L. BMA results showed that granulocyte cells and erythroid cells accounted for 54.2% and 25.0%, respectively. At 31 months after cell infusion, HGB was 134 g/L, and the patient’s condition continued to be stable.

## Discussion

Several risk factors have been found for PRCA after HSCT, mainly including ABO blood group incompatibility (especially in blood group A donor to blood group O recipient, with IH titer higher than 1:16) and sibling donor (4, 11, 16, 17). In our study, all the patients were blood group O, with IH titers above 1:16, while the blood group of donors were 1 case of A and 2 cases of B. And all the 3 cases received sibling HSCT. In terms of risk factors for PRCA occurrence, our study is consistent with previous reports.

Regarding the treatment of PRCA after HSCT, with supportive RBC transfusion, there is a high probability of spontaneous remission after 1∼2 months or so. But beyond 2 months, other treatments would be required (3, 9, 18, 19). Immunosuppressive therapy, such as CsA is currently the first-line choice, with an effective rate of 65%-87% (19–22). For patients unresponsive to CsA, other regimens such as tacrolimus, rituximab, plasma exchange, and DLI have been reported, but the efficacy was controversial among centers (10, 12). In our study, all three cases were treated with immunosuppressive therapy and RBC transfusion after the diagnosis of PRCA, but the result was not significantly effective, patients are still dependent on RBC transfusion. So we tried new treatments of cell therapy.

To our knowledge, several studies of using UCB and MSCs to treat acquired PRCA have been reported, however, cases used in PRCA after HSCT are rare (13, 15, 23–25). Two cases were reported using MSCs derived from umbilical cord and adipose to treat PRCA after ABO-mismatched allo-HSCT, and patients reached rapid recovery without any side effects (24, 25). One study used UCB to treat 4 patients with PRCA after allo-HSCT, but the efficacy was not ideal, which might because immunosuppressive agents were not allowed from 2 weeks before to 2 months after UCB infusion (23). In our study, with conventional therapy ineffective, we explored three regimens of UCB and MSCs in combination with immunosuppressive agents and RBC transfusion to treat PRCA after HSCT, all patients achieved significant progress and RBC transfusion independence. This suggests that the combination of cell infusion can effectively improve the therapeutic effect of PRCA after HSCT when conventional treatment is ineffective.

RBC transfusion independence is a key indicator of red blood cell engraftment (8, 10), therefore, we discussed the mechanism of HSCT combined with cell therapy based on this indicator in our study and previous literatures. As shown in **Table 1**, the time of RBC transfusion independence was 2 weeks (Case 2, 3 doses of UCB-MNC), 2 months (Case 1, UCB combined with MSC), and 3 months (Case 3, UCB), respectively. That might related to cell source and dose, which are vital to the efficacy of cell therapy (26). In Case 1, HSCT combined with UCB and MSCs might play a synergistic role in hematopoietic supporting and immune regulation, which displayed a better effect than in Case 3. There are hematopoietic stem cells (HSC), MSCs, other progenitor cells, and immune cells in UCB, which could perform hematopoiesis support and immune regulation function (27, 28). A recent study showed that after UCB infusion in AML patients, immune functions were enhanced by promoting cell proliferation and cytokine secretion of CD8+T cells and natural killer cells and mitigating the immunosuppressive effects of CD14+ monocytes (29). MSCs play a remarkable immunomodulation role by releasing cytokines, growth factors, chemokines, and exosomes through paracrine actions, such as vascular endothelial growth factor (VEGF), and hepatocyte growth factor (HGF) (30, 31). MSCs could regulate the immune function by modulating the activation, expansion, and differentiation of natural killer cells, dendritic cells, B lymphocytes, macrophages, and T lymphocytes (32–34). Multiple doses of UCB-MNC infusion have been proven to enhance the effect of stem cells and improve disease outcomes (35–40). In our report, 3 doses of UCB-MNC were more effective than one unit of UCB, which conformed to previous studies.

**Table 1:** the information of patient, stem cells and key outcomes of 3 patients.

Patient	Case 1	Case 2	Case 3
age	32	38	36
sex	female	female	female
date of HSCT	November 2018	September 2023	November 2018
anti-A/anti-B IgM titers	1:64 and 1:128	1:128 and 1:128	1:32 and 1:16
date of PRCA diagnosis	July 2019	January 2024	February 2019
treatments administered	RBC+ UCB+UC-MSC	Tacrolimus+RBC+ UCB-MNC	Methylprednisolone+ tacrolimus+ UCB
stem cell number	UCB:20.83×10^8^; MSC:6.0×10^7^	5.0×10^7^/dose, 3 doses	4.51×10^8^
time of RBC transfusion independence	two months after cell infusion	two weeks after cell infusion	three months after cell infusion

HSCT, hematopoietic stem cell transplantation; PRCA, pure red cell aplasia; RBC, red blood cell; UCB, umbilical cord blood; UCB-MNC, umbilical cord blood mononuclear cells; UC-MSC, umbilical cord mesenchymal stem cells.

However, our study has some limitations, which are mainly from the inherent shortcomings of small-scale sample cases and retrospective studies. A well-designed prospective research with controls and more studies are needed to confirm our results and the exact mechanism of cell therapy. This report would provide supportive data for PRCA treatment, reduce patients’ blood transfusion dependence, and improve the quality of life. Further studies on different cell combinations and doses of UCB, UCB-MNC, and MSCs are deserved to evaluate the efficacy of different treatment regimens.

## Conclusion

This report provides three cell therapy strategies using UCB and UC-MSCs for the treatment of PRCA after allo-HSCT. Our study demonstrates the safety and efficacy of 3 doses of UCB-MNC regimen and UCB combined with UC-MSCs regimen, providing a new treatment option for patients with PRCA after allo-HSCT.

## Data Availability

The original contributions presented in the study are included in the article/supplementary material. Further inquiries can be directed to the corresponding author.
